# Cross-Band Coupling of EEG Infraslow Oscillations during NREM Sleep in Mice

**DOI:** 10.21203/rs.3.rs-10985934/v1

**Published:** 2026-10-05

**Authors:** Max Tan, Oscar Ramirez-Plascencia, Wei Wang, Amanda Studnicki, J. Kent Werner, Elizaveta Tremsina, Shiqian Shen, Can Zhang, Tingying Helen Zeng, Robert Thomas, Haoqi Sun

**Affiliations:** Beth Israel Deaconess Medical Center; Beth Israel Deaconess Medical Center; Massachusetts General Hospital; Uniformed Services University; Uniformed Services University; Massachusetts Institute of Technology; Massachusetts General Hospital; Massachusetts General Hospital; InnoBridge Institute; Beth Israel Deaconess Medical Center; Beth Israel Deaconess Medical Center

**Keywords:** infraslow oscillation, infraslow fluctuation, sleep, EEG, electrophysiology, cross-band coupling

## Abstract

The infraslow oscillation (ISO) is a ~ 0.02 Hz rhythmic fluctuation in electroencephalographic (EEG) power during NREM sleep. Prior studies were limited to sigma-band ISO, linked to rhythmic norepinephrine release in mice. Whether ISOs exist in other bands, and how ISOs across bands are coupled, remain unknown. We analyzed cross-band ISO coupling across six frequency bands during NREM using a wild-type (WT) discovery dataset of 122 recordings from 72 mice and WT validation of 7 mice. A transgenic (TG) validation of 6 mice was added to test ISO coupling’s robustness to Cre knock-in on the LepR gene (LepR-Cre). Band power time series were filtered to the ISO range of 0.01–0.03 Hz. Cross-band coupling was assessed using Spearman’s correlation and phase-locking value (PLV) across all 15 band pairs. ISOs were strongest in alpha, sigma, beta, and theta bands, weak in gamma, and absent in delta. Alpha, sigma, beta, and theta ISOs formed a highly correlated motif with replication in WT and TG (r = 0.61–0.77), while gamma had moderate correlation (r = 0.30–0.42), and delta was uncorrelated (p > 0.05). All band pairs showed significant phase locking with replication (FDR-corrected q < 0.01), with stronger consistency in alpha, sigma, theta, and beta (PLV 0.54–0.71), and weaker consistency involving delta and gamma (PLV 0.12–0.37). EEG ISOs extend beyond the sigma band to alpha, beta, and theta bands, forming a strongly coupled motif that may reflect a fundamental organization principle of sleep microstructures at a minute scale.

## INTRODUCTION

Infraslow oscillations (ISO, or infraslow fluctuation, ISF) exist in non-rapid eye movement (NREM) sleep in both humans^1^ and animals^2,3^. ISO is a close-to-50-second fluctuation (about 0.02Hz) in the spectral power time series of electroencephalography (EEG) within a frequency band. The sigma-band ISO is increasingly viewed as a fundamental organizing rhythm of the sleeping brain^4,5^ at a minute scale, where spindles preferably occur during the falling phase of NE ISO that favors memory consolidation^6,7^, and arousals occur at the rising phase of NE ISO^8^, leading to an ISO in the alpha band as well. From a neurotransmitter perspective, the sigma-band ISO is related to a pulsatile release of norepinephrine (NE, noradrenaline) from the locus coeruleus (LC)^9^. From a functional perspective, the sigma-band ISO is also related to glymphatic clearance^10^, which is supported by rhythmic vasomotion^11^. NE has multiple functions, including vasoconstriction^12^, modulation of neural activity^13^, wake promotion^14^, and cognitive enhancement^15^. Therefore, the ISO represents an important electrophysiological pattern that reflects neurotransmitters and functions, and links to brain health and diseases.

However, previous descriptions of ISOs have been restricted to the sigma band, where spindles occur. Kjaerby et al.^16^ discovered coupled infraslow oscillations across five neuromodulators during NREM sleep in mice, including serotonin, dopamine, histamine, NE, and acetylcholine, suggesting that the ISO may reflect a broad neuromodulation pattern rather than one specific to spindles or arousals. Specifically, Turi et al^17^ identified serotonergic modulation of infraslow oscillations in the dentate gyrus in mice. These animal studies support the idea that different neurotransmitters, when coupled, are associated with brain oscillations across different frequency bands. Therefore, we hypothesize that there is also cross-band coupling among ISOs in mice. Further, we studied whether such ISO coupling persists in a model of sleep-wake regulation impairment using transgenic (TG) mice with a leptin receptor mutation. Therefore, studying the cross-band coupling among ISOs is important since its impairment could reflect impairment in the fundamental organization of sleep in humans, hence linked to insomnia, compromised immune/hormone/inflammation response^18^, cardio-/cerebro-vascular diseases^19,20^, and neurodegenerative diseases^21^.

Here, we quantified the cross-band coupling of ISOs across six EEG frequency bands during NREM sleep in both wild-type (WT) and transgenic mice (TG, LepR-Cre) to test ISO coupling’s robustness to Cre knock-in on the LepR gene. We measured coupling based on amplitude correlation and phase locking. The results confirm the cross-band coupling among ISOs and provide hypotheses about the mechanism of ISOs across multiple bands. ISO is not a band-specific phenomenon, but rather a brain-wide modulation expressed across multiple coupled bands.

## RESULTS

### Alpha-, sigma-, beta-, and theta-band ISO PSDs showed a single-peak pattern

As detailed in Methods, the wild-type (WT) discovery dataset was the Mouse Sleep Staging Validation (MSSV) dataset^22^, consisting of 72 healthy mice (male 52, 72%) with 122 recordings across five independent laboratories after excluding transgenic and unknown-genotype animals. The WT validation dataset was the Oxford Mouse Polysomnography Benchmark Data Set (OxfordBenchmark)^23^ consisting of 7 adult male C57BL/6 WT mice. The TG validation dataset consisted of 6 adult male LepR-Cre mice^24^ (JAX stock #032457).

Figure 1 shows the averaged ISO power spectral densities (PSDs) across all subjects per dataset and band, with males and females as separate strata in the MSSV dataset. Four bands, including alpha (8–12 Hz), sigma (11–15 Hz), beta (15–30 Hz), and theta (5–10 Hz), showed single-peak morphology (visually assessed) across datasets, while the females in MSSV had less clear single-peak morphology. In MSSV males, the frequency peak was 0.0166 Hz for alpha, 0.0161 Hz for sigma, 0.0154 Hz for theta, and 0.0187 Hz for beta. The gamma band showed single-peak morphology in two datasets/strata: the WT discovery dataset (MSSV) male stratum and the WT validation dataset (OxfordBenchmark). In contrast, the delta band (1–4 Hz) did not show a single-peak pattern on visual inspection.

Alternatively, after removing the aperiodic component using FOOOF (fitting oscillations & one over f), the peak around 0.02Hz in the ISO PSD became much attenuated, as shown in Supplementary Figure S1. Only the sigma- and alpha-band ISO PSDs from the LepR-Cre TG validation dataset showed a broad peak around 0.02Hz. Therefore, we did not proceed with cross-band coupling analyses for this alternative exploration.

### Alpha-, sigma-, beta-, and theta-band ISOs were coupled based on Spearman’s correlation

Figure 2. Cross-band coupling of EEG ISOs based on the results that were replicated in both WT and TG validations. Each panel is a network with six vertices representing the ISO bands, with edges encoding a coupling metric for each band pair from the WT discovery dataset. Only the replicated significant band pairs are shown (BH-FDR-corrected q < 0.05). Edge color follows a zero-centered cool-warm scale (blue negative, red positive) and edge width scales with the magnitude of the coupling. (a) Spearman's correlation. Edges show Spearman’s correlation. The vertex positions come from a 2-dimensional multidimensional scaling (MDS) embedding of the dataset-averaged correlations, so that strongly coupled bands are placed close together. Alpha, sigma, theta, and beta form a tightly coupled cluster; gamma is more distant, and delta is isolated with no significant edges. (b) Phase-locking value (PLV). Edges show the PLV between the two bands' ISO phases. Vertices are arranged on a phase clock with the counter-clockwise direction indicating phase lead. Each band's angular position is its relative ISO phase with respect to the first eigenvector of the complex phase-coupling matrix. The result showed an alpha-beta-sigma-theta phase order among the ones with higher PLV.

In replication, the WT discovery dataset (MSSV) had 11–15 significant (BH-FDR-corrected q < 0.04) band pairs across five labs; 11 were common and had the same sign across all five labs, except delta-gamma, delta-beta, delta-sigma, and delta-alpha. In the WT validation dataset (Oxford Benchmark), 10 out of 11 band pairs were replicated (BH-FDR-corrected q < 0.017), with delta-theta dropped (q = 0.38). In the TG validation dataset, 11 band pairs were replicated (q < 0.031), including delta-theta. In terms of effect size, the Spearman’s correlations remained similar for WT discovery vs. WT validation (Fig. 3a), supporting replication in WT. The Spearman’s correlations were also similar for WT validation vs. TG validation (Fig. 3e), supporting agreement between WT and TG (Lep-Cre). In contrast, the Spearman’s correlation was on average 0.11 [0.08–0.15; p < 0.001] higher in the TG validation (Fig. 3c) compared to WT discovery. In sensitivity analyses, first z-transforming the band power time series within each NREM bout led to a minimal reduction in effect sizes of less than 0.011 and no change in the ranking or statistical significance among band pairs, reflecting no effect of between-bout differences. Another alternative approach of averaging per-bout coupling metrics instead of concatenating the NREM bouts also had a minimal change in the effect sizes, ranging from − 0.024 to + 0.010, and no change in the ranking and statistical significance among band pairs.

### Alpha-, sigma-, beta-, and theta-band ISOs were coupled based on Phase Locking Value

In Fig. 2b, the phase locking value (PLV) network has a consistent structure with Spearman’s correlation in Fig. 2a, with alpha, sigma, theta, and beta having strong phase-lagging consistency among them (0.54–0.71 in WT discovery), while the phase relationship is less consistent for delta and gamma with other bands (0.12–0.37 in WT discovery).

In replication, the WT discovery dataset had all band pairs significant (BH-FDR-corrected q < 0.031) across five labs, and these results replicated in both WT (q < 0.016) and TG (q < 0.031) validation datasets. In terms of effect size, the PLVs remained similar for discovery vs. WT validation (Fig. 3b), again supporting replication in WT. Meanwhile, the PLVs were higher in the TG validation compared to WT discovery (0.03 [0.01–0.05; p = 0.015] higher, Fig. 3d) and to WT validation (0.06 [0.02–0.10; p = 0.008] higher, Fig. 3f). In terms of sensitivity analyses, averaging per-bout coupling metrics rather than concatenating NREM bouts yielded higher PLV, with a range of [+ 0.01, + 0.15] across all band pairs, indicating stronger within-bout coupling than the global concatenation approach. Again, the ranking and statistical significance did not change across band pairs.

### Visualizations

In Fig. 4, we show an example of a 400-second band power time series, band-pass filtered at 0.01-0.03Hz, from the WT discovery dataset (MSSV), with Spearman’s correlation and PLV closest to the MSSV cohort mean (from mouse subject sub-087, WT male adult, 12 hours of recording). The visual findings were consistent with Fig. 2, in which alpha, sigma, theta, and beta are closely coupled and rise and fall together in a ~ 50-second rhythm (marked by gridlines). Meanwhile, gamma ISO was less coupled, and delta ISO was not coupled to any other ISOs. Both gamma and delta ISOs had a less obvious ~ 50-second rhythm.

In Fig. 5, we show a zoomed view of 100 seconds, along with two further zoomed-in EEG signals, each 20 seconds long. The EEG traces confirm changes in the 0.01-0.03Hz band-pass-filtered band-power time series, where the 50-second fluctuation is harder to capture due to excessive compression of the EEG signals at that scale. In the Supplementary Video, we show a dynamic illustration of 2 minutes of EEG played at 2x real time, with a dynamic Spearman’s correlation network based on the current 200-second EEG, and each band's instantaneous ISO phase as a rotating node on a phase circle with links indicating the PLV.

## DISCUSSION

Our results provide new evidence that ISOs occur in the alpha, theta, and beta bands beyond the known sigma band, and are closely coupled among them during NREM sleep. They rise and fall together on a minute scale. This minute-scale rhythm becomes a new member of the multi-scale oscillation family of brain^25^, biological, and environmental rhythms, spanning from 1000Hz for neuronal action potentials to circadian rhythms, seasons, and beyond. The ISOs and their coupling may represent the fundamental organization of sleep, and their impairment could drive disease progression.

The mechanisms underlying alpha- and beta-band ISOs may overlap with those of the sigma-band ISO, reflecting infraslow fluctuations in norepinephrine (NE) from the locus coeruleus (LC), a wake-promoting neurotransmitter. Therefore, arousals (alpha and beta bands) are more likely to occur in the rising phase of NE release^8^, and spindles are more likely to occur in the falling phase of NE release^6,7^, leading to coupled ISOs across alpha, beta, and sigma bands as in Fig. 2.

The mechanism of theta-band ISO is unknown. One possibility is related to serotonin release: in mice, serotonin suppresses cortical theta bursts, and a low serotonin state is essential for theta burst-related hippocampal activity during NREM sleep^17^. Meanwhile, Kjaeby et al.^16^ have discovered that all five neurotransmitters, including serotonin, exhibit synchronized infraslow oscillations during sleep in mice. Taken together, these two studies suggest that theta’s coupling with alpha, beta, and sigma may be related to infraslow oscillations in serotonin release. An electrophysiological explanation is supported by the existence of a temporal sequence: brief theta bursts tend to occur just before the downstate of the slow oscillation, whereas sleep spindles preferentially occur afterward, during the down-to-up transition and subsequent upstate^26,27^, therefore contributing to the coupling of theta-band ISO with others.

The gamma band showed weak ISO as in Fig. 1f, which could be a residual effect of the beta band, since the band frequency boundaries are arbitrary. The gamma oscillation is usually implicated in active thinking and sensation binding. Meanwhile, it also occurs during NREM sleep. Previous studies have demonstrated that gamma oscillations are coupled to slow wave (0.5-3.5Hz, in our case, closest to the delta band at 1-4Hz) phases, similar to spindles, in both mouse experiments^28^ and human observations^29^.

We did not find ISO in the delta band as in Fig. 1a. This is in contrast to ISO of slow wave activity reported in rats^30^ and community-dwelling humans^31^. It is possible that periods of sleep with strong slow wave activity in the delta band without ISO are required, with distinct non-overlapping functional effects to ISO. In human clinical data, pathological sleep states are dominated by an overabundance of oscillations near the range of the ISO, such as periodic breathing and limb movements^32^, while delta power-rich sleep (usually stable NREM sleep) is relatively resistant to fragmentation^33^.

The coupled EEG ISOs could serve as a temporal organization principle for various sleep microstructures and their functions at a minute scale, analogous to cortical columns^34^ as a spatial organizational principle of the cortex^35^. One example in mice is that the sigma-band and alpha-band ISOs organize the timing of memory replay and consolidation^5,36^. Another example in humans is that the multi-band ISOs can be manifested as cyclic alternating pattern (CAP)^7^. During CAP, the A phases occur between 10–60 second intervals, falling into the infraslow range. There are A1, A2, and A3 sub-phases under the A phase, where A1 contains K-complexes and spindles (sigma-band ISOs), and A3 contains arousals (alpha- and beta-band ISOs). CAP has traditionally been thought of as an instability marker that organizes NREM into stable and unstable states^33^. Recent evidence supports the hypothesis that a certain amount of unstable sleep is needed in normal physiology^8,11,21^. CAP also has multi-organ implications, in which the autonomic nervous system in the body is coupled with EEG CAP in the brain^7,37^, forming a multi-organ infraslow oscillation (MISO)^38^. However, in humans, the cross-band coupling of ISO in EEG or other physiological modalities has not yet been described, but can be predicted to occur.

Our choice of a LepR-Cre TG validation dataset was to test ISO coupling’s robustness to Cre knock-in at the LepR locus. In the brain, leptin is involved in the integrative circadian control of eating and energy homeostasis^39,40^. The results in Fig. 3 showed the same statistically significant band pairs as in WT, indicating successful replication. However, the TG validation had slightly larger coupling strengths, which could be explained by NREM bout duration as follows. The mean NREM bout duration was 3.4 minutes for TG validation, which was longer than WT discovery at 1.7 minutes (ANOVA followed by Tukey’s test, p < 0.001) and WT validation at 1.5 minutes (p < 0.001). Meanwhile, WT discovery and WT validation had similar NREM bout durations (p = 0.68), excluding the impact of differences in age and sex distributions. The longer NREM bout duration in the TG validation could be due to automated sleep staging, which tends to produce sleep stages with fewer transitions. Along this line, more TG animal models should be tested, such as mouse models with neurodegenerative or amyloid pathologies, which is a valuable next step given the link between ISO, glymphatic clearance, and amyloid pathology.

The clinical relevance of EEG ISOs in multiple bands and their cross-band coupling should be established in humans. Our findings may help understand the complex coordination among multiple neurotransmitters, each with a unique vasomotor profile, potentially contributing to sleep disturbances, the regulation of glymphatic clearance^41^, and associated with plasma- or cerebrospinal fluid-based biomarkers^42^. Therefore, the impairment of ISOs and their coupling may have downstream consequences such as insomnia, pain^43^, depression, Alzheimer’s disease^10^, cardio-metabolic diseases^44^, and developmental delay^31^. Epidemiological and prospective association studies of multi-band/cross-band ISOs with incident health outcomes, stratified by age and sex, should be conducted, such as those done with CAP^21^. The effect of various sleep-targeted treatments on multi-band/cross-band ISOs should be evaluated, including continuous positive airway pressure (CPAP)^45^, auditory/magnetic/electric stimulations, and gut microbiome^46^ approaches.

Our study has limitations. First, we did not consider the stable/unstable states during NREM sleep, which are more established in humans^33,47^ but could still exist in mice. Having a metric to quantify these states and focusing only on the unstable states may lead to a more regular ISO and, hence, a stronger peak in the spectrum. Second, it is unknown whether EEG ISO also occurs in REM sleep, given that acetylcholine also exhibits an infraslow rhythm^16^. It is possible that phasic rapid eye movement episodes occur in the infraslow frequency range in humans. Other electrophysiological patterns in human REM sleep, such as sawtooth waves^48^ or iota oscillations (25–35 Hz)^49^, could also be investigated in the context of ISO. Third, we didn’t examine cross-modal ISO, including heart rate^50^, breathing stability^51^, respiratory tidal volume fluctuations^52^, pupil size^53^, and stomach electricity^54^. Lastly, a larger sample size is needed to facilitate the study of age- and sex-dependence. We observed an ISO peak only around 0.02Hz in males, but not in females (Fig. 1), which could be related either to the smaller female sample size or to inherent sex-dependent differences in NREM sleep, analogous to those in humans^62,52,55^.

In summary, the characterization of cross-band coupling of EEG ISOs during NREM sleep in mice provides a starting point for a more comprehensive understanding of ISOs. The physiology could be a fundamental organizing principle of sleep at the minute scale.

## METHODS

### Dataset

The wild-type (WT) discovery dataset was the Mouse Sleep Staging Validation (MSSV) dataset^22^, which is publicly available from OpenNeuro (ds006366, version 1.0.1). It contains a frontal EEG and EMG recordings from 92 healthy mice with 148 recordings across five independent laboratories. After excluding transgenic and unknown-genotype animals, the WT discovery dataset consisted of 72 mice with 122 recordings, with 32, 17, 10, 7, and 6 mice across source laboratories. Each 4-second epoch was manually labeled by the experimenter as wake, NREM, REM, or artifact. The EEG sampling frequency was 128Hz. The dataset was predominantly male (52/72, 72%). Based on age classification, 22 recordings (18%) were obtained from juvenile mice (≤ 3 months), 92 (75%) from adults (3–18 months), and 8 (7%) from old mice (≥ 18 months). The recording duration varied across five labs, with an average length ranging from 3 hours to 3 days. As described in Rose et al.^22^, all animal studies were approved by the respective laboratory’s national authorities and carried out according to ethical guidelines (European Communities Council Directive (86/609/EEC)) and ARRIVE (Animal Research: Reporting In Vivo Experiments).

The WT validation dataset was the Oxford Mouse Polysomnography Benchmark Data Set (OxfordBenchmark)^23^, which is publicly available from the National Institute of Allergy and Infectious Diseases Data Ecosystem and Zenodo (record 10200482, version 1.0.0). We used 7 adult male C57BL/6 WT mice from the “pilot” (1 mouse) and “test” (6 mice) partitions. It contains one-channel EEG and EMG recordings. Each 4-second epoch was manually labeled by at least 4 scorers, and we used their consensus. The EEG sampling frequency was 256Hz. The recording duration was 24 hours in 6 mice and 12 hours in 1 mouse. As described in Brodersen et al.^23^, the Animal Welfare and Ethical Review Body at the University of Oxford granted ethical approval for the animal experimentation. All electrophysiological recordings were performed in accordance with the United Kingdom Animal Scientific Procedures Act 1986 under personal and project licenses granted by the United Kingdom Home Office.

The TG validation dataset consisted of 6 adult male LepR-Cre mice^24^ (JAX stock #032457). EEG recordings were done as part of a series of studies of wake-sleep recordings in mice. Mice were implanted with four skull screws attached to a 6-pin connector for EEG/EMG, and bilateral optical fibers 0.5 mm above the dorsomedial hypothalamic area. We used the baseline sleep periods, totaling 4 days per mouse. Since manual sleep stages were not available for all but one mouse, we trained a U-Sleep sleep staging deep learning model^56^ on the MSSV dataset using the EEG channel and automatically scored each 4-second epoch. Manual sleep stages were available for 3 days in one mouse, which was used to report U-Sleep sleep staging performance as a sanity check. The EEG sampling frequency was 512Hz. The average recording duration was 6.7 days (standard deviation: 0.6 days, min: 6 days, max: 7.3 days). In the one mouse with manual sleep stages available for 3 days, the U-sleep automatic sleep staging accuracy for Wake, NREM, and REM was 68.5%, 79.3%, and 35.8%, respectively, with an overall Cohen’s kappa of 0.48 and macro F1-score of 0.61. All procedures were performed with formal approval of our protocols from the Institutional Animal Care and Use Committee at Beth Israel Deaconess Medical Center. All precautions were taken to minimize pain and discomfort in the mice in accordance with the National Institutes of Health Guide for the Care and Use of Laboratory Animals.

### EEG Infraslow Oscillation Quantification

The analysis was based on the approach in Fernandez et al.^57^ The same preprocessing pipeline was applied to all datasets. For each 4-second epoch, the power spectral density (PSD) (no padding since the number of sample points is a power of 2) was estimated using the multitaper method^58,59^ (7 tapers, bandwidth 2.0 Hz) over 1–45 Hz, with the upper bound chosen to exclude the minimum AC line frequency of 50 Hz, since the recordings were of international origin. Six EEG frequency bands were analyzed: delta (1–4 Hz), theta (5–10 Hz), alpha (8–12 Hz), sigma (11–15 Hz), beta (15–30 Hz), and gamma (30–45 Hz). The upper bound of the theta band was extended to 10 Hz to reflect the characteristically higher theta frequency observed in rodents compared with humans^60^. Band power was computed as the area under the PSD curve between the band's lower and upper frequencies using the trapezoidal rule. Then the band powers were normalized to the within-subject average band power across all NREM epochs, which exclude epochs within 4 seconds (1 epoch) around stage transitions. For each band, this produced a time series of band power sampled at 0.25 Hz, ensuring the same sampling frequency across datasets.

Band power time series within NREM bouts lasting ≥ 96 seconds, which covers approximately 2 ISO cycles, were retained for analysis. To obtain ISO PSD, for each band, the PSD of the band power time series from each NREM bout was estimated using Welch's method with a Hamming window spanning the full length of the time series and a frequency range of 0.005–0.1 Hz (Nyquist frequency is 0.25/2 = 0.125Hz). Welch’s method was used because the band power time series were too short to support reliable multitaper spectral estimation. The ISO PSD was then normalized by its total power between 0.005-0.1Hz. Because NREM bouts vary in duration, the overall ISO PSD was obtained by first linearly interpolating onto the frequency grid of the longest bout and then averaging across bouts.

We also explored repeating the same analysis by removing the aperiodic spectral (1/f) component using fitting oscillations & one over f (FOOOF)^61^, which helps account for gradual changes in aperiodic activity and overnight slow-wave activity reduction related to synaptic homeostasis, thereby creating artificial correlations that reflect circadian or other signal drift.

### Cross-Band EEG Infraslow Coupling Analysis

Each EEG band power time series was band-pass filtered between 0.01 and 0.03Hz before dividing into NREM bouts. Two coupling metrics were computed from filtered band power time series across the bands. (1) Spearman’s correlation was computed based on the filtered band power time series after concatenating across NREM bouts within the same recording, from the 15 band pairs (no order), providing a measure of monotonic amplitude co-variation. (2) Phase-locking value (PLV)^62^ was computed as the magnitude of the mean complex phase difference for the 15 band pairs, providing a measure of phase lag consistency. The instantaneous phases were first obtained from the complete band power time series using the Hilbert-Huang transform, divided into NREM bouts, and then concatenated across bouts within the same recording. There were two sensitivity analyses: (1) z-transform within each NREM bout before concatenation for Spearman’s correlation metric; and (2) average per-bout coupling metrics instead of the concatenation for both Spearman’s correlation and PLV metrics.

### Statistical Analysis

We averaged the cross-band coupling metrics for multiple recordings within the same subject. To obtain an overall cross-subject p-value for each band pair and coupling metric, each coupling metric was transformed to a scale with a null value of 0: Spearman’s correlation was Fisher z-transformed; and PLV was z-transformed according to the mean and standard deviation of 2000 bootstraps. Then, the cross-subject p-value was obtained using a two-sided 1-sample Wilcoxon signed-rank test against 0.

The WT discovery dataset (MSSV) was used to discover candidate cross-band coupling metrics based on (1) false discovery rate (FDR) control using Benjamini-Hochberg (BH) in every single source laboratory; and (2) having the same effect sign across the five source laboratories. The significant cross-band coupling metrics (BH-corrected p < 0.05) were then validated in the WT validation dataset (OxfordBenchmark) to confirm replication, using BH correction based on the number of discovery-significant metrics. The TG validation dataset is used to explore the differences in significant ISO coupling metrics compared to the replicated metrics in the WT validation dataset.

All analyses were done using Python 3.10.12. Code and package versions are available as mentioned in the Code Availability section.

## Supplementary Material

This is a list of supplementary files associated with this preprint. Click to download.

• supplISOcoupling2.docx

## Figures and Tables

**Figure 1:**
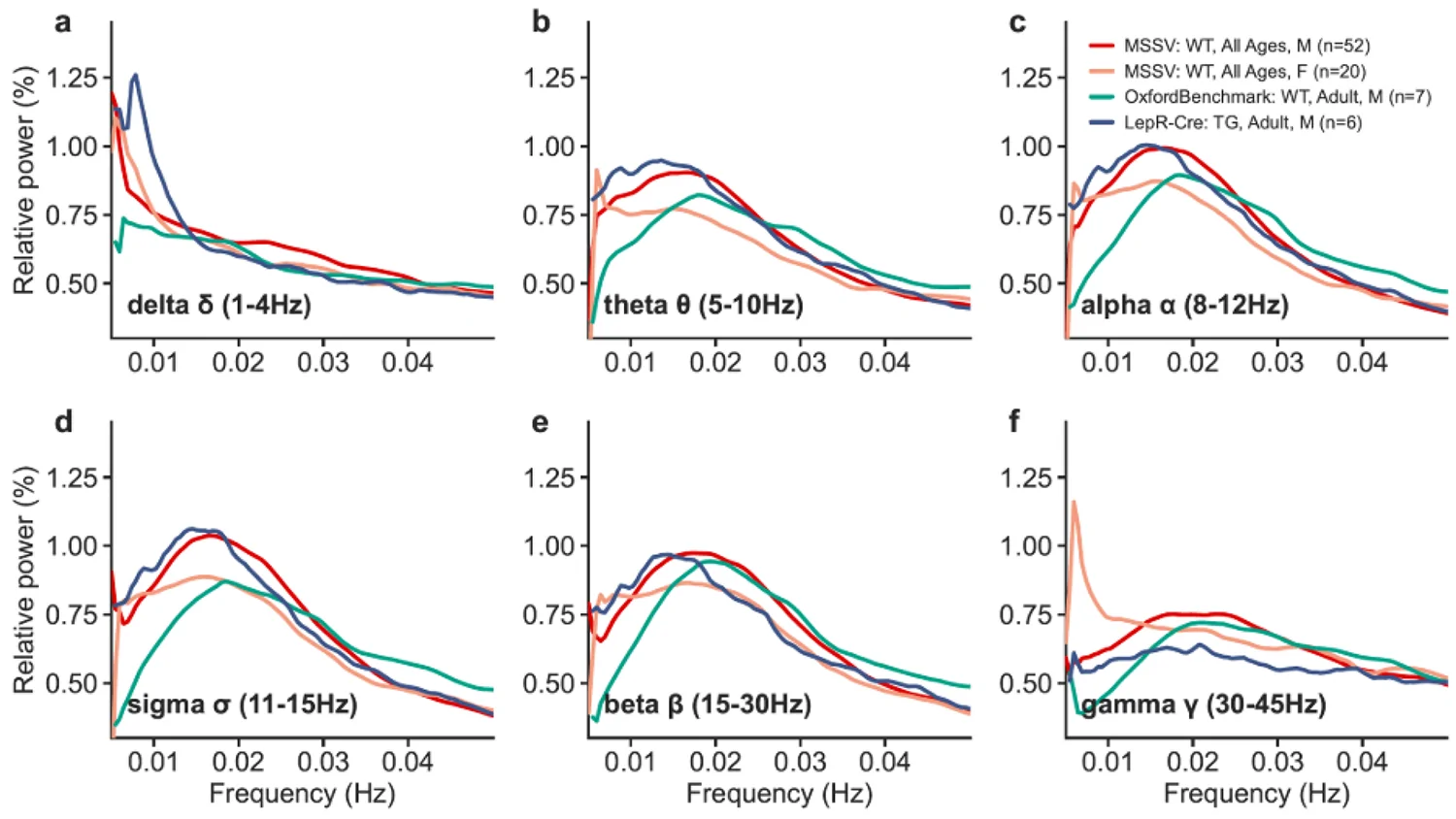
EEG ISO PSD per frequency band, averaged across all subjects per dataset and band, with males and females as separate strata in the MSSV dataset. The relative power (%) of the ISO PSD is shown after normalization by its total power between 0.005-0.1Hz.

**Figure 2:**
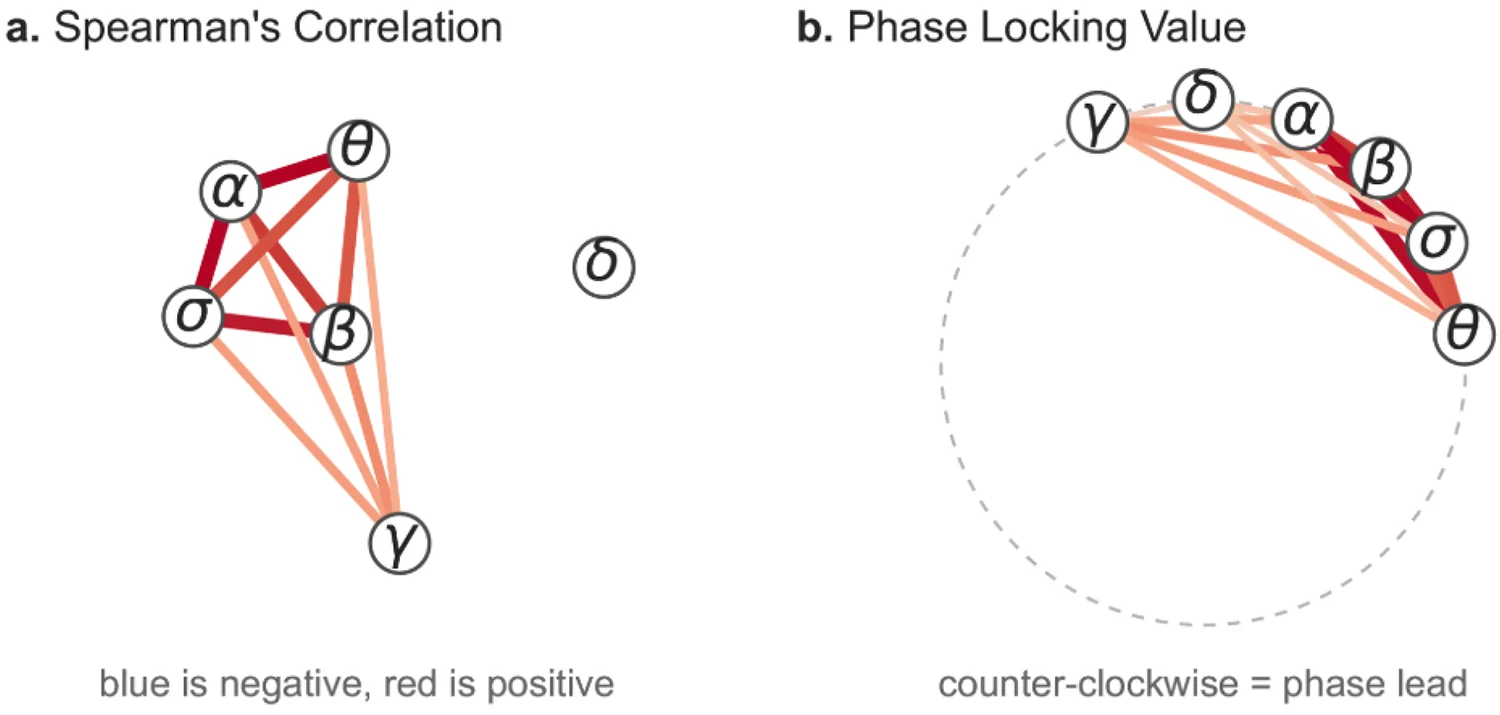
Cross-band coupling of EEG ISOs based on the results that were replicated in both WT and TG validations. Each panel is a network with six vertices representing the ISO bands, with edges encoding a coupling metric for each band pair from the WT discovery dataset. Only the replicated significant band pairs are shown (BH-FDR-corrected q<0.05). Edge color follows a zero-centered cool-warm scale (blue negative, red positive) and edge width scales with the magnitude of the coupling. (a) Spearman's correlation. Edges show Spearman’s correlation. The vertex positions come from a 2-dimensional multidimensional scaling (MDS) embedding of the dataset-averaged correlations, so that strongly coupled bands are placed close together. Alpha, sigma, theta, and beta form a tightly coupled cluster; gamma is more distant, and delta is isolated with no significant edges. (b) Phase-locking value (PLV). Edges show the PLV between the two bands' ISO phases. Vertices are arranged on a phase clock with the counter-clockwise direction indicating phase lead. Each band's angular position is its relative ISO phase with respect to the first eigenvector of the complex phase-coupling matrix. The result showed an alpha-beta-sigma-theta phase order among the ones with higher PLV.

**Figure 3:**
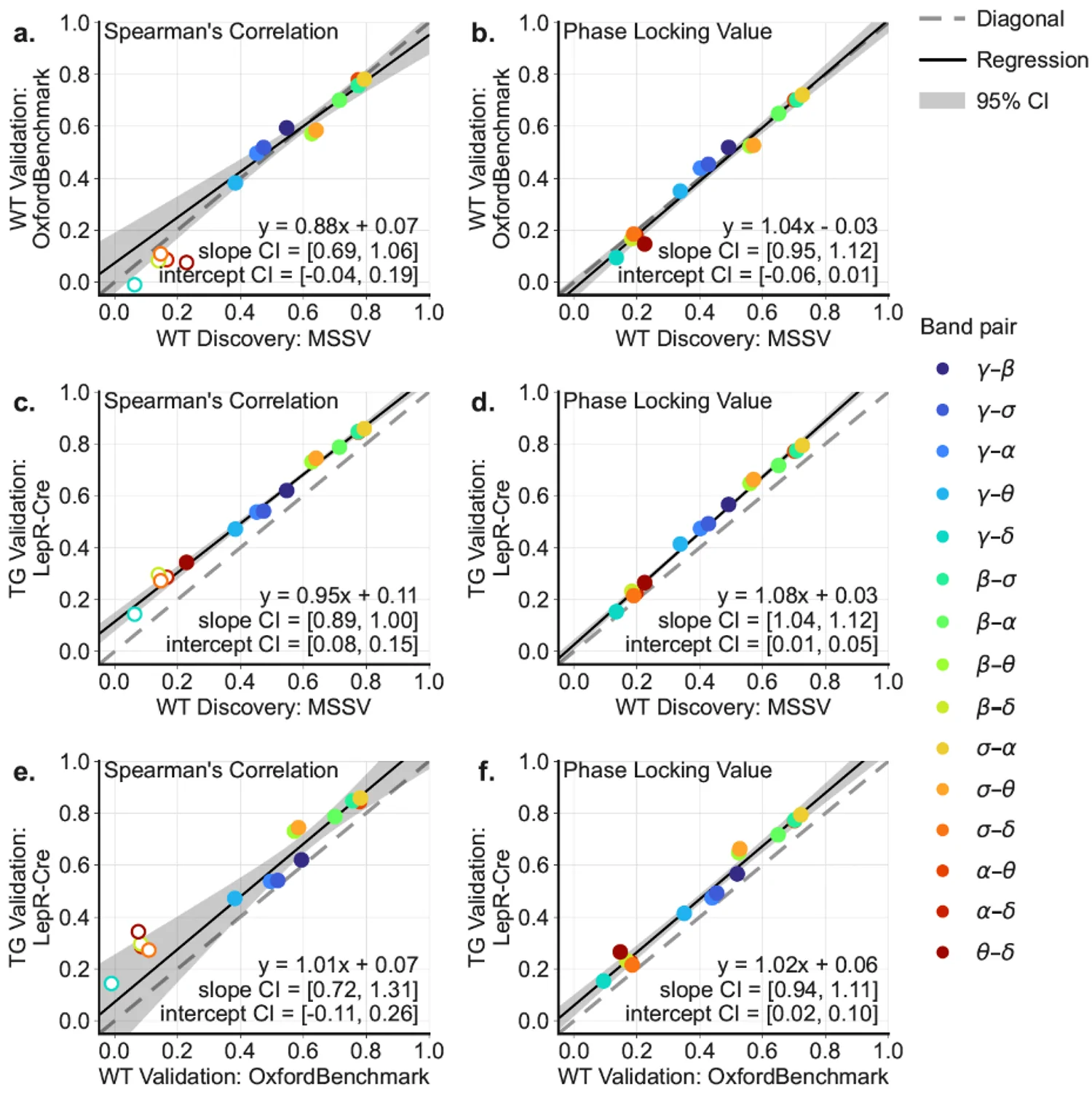
Reproducibility of cross-band ISO coupling effect sizes between datasets. Filled solid points denote band pairs that remained statistically significant after Benjamini-Hochberg FDR correction (q < 0.05); open points did not reach significance. For panels comparing the WT discovery dataset (MSSV) to a validation dataset (a-d), significance is defined in the validation dataset, i.e., discovery findings that were reproduced. For panels comparing the two validation datasets directly (e-f), significance requires FDR significance in both validation datasets. The gray dashed diagonal marks the identity line, i.e., perfect agreement between datasets. The solid black line is the linear regression fit through the significant (filled) points, with the shaded gray band showing its 95% confidence interval (CI); the regression equation and the 95% CIs for slope and intercept are also reported. (a) Spearman's correlations between ISOs of all band pairs from WT discovery (MSSV) vs. WT validation (OxfordBenchmark). The slope CI contained 1, and the intercept CI contained 0, indicating good replication. (b) PLVs between ISOs of all band pairs from WT discovery vs. WT validation. The slope CI contained 1, and the intercept CI contained 0, indicating good replication. (c) Spearman's correlations from WT discovery vs. TG validation (LepR-Cre). The slope CI contained 1, but the intercept CI did not contain 0, indicating a uniformly higher cross-band ISO coupling in terms of Spearman's correlation in TG LepR-Cre validation compared to WT discovery. (d) PLVs from WT discovery vs. TG validation. The slope CI did not contain 1, and the intercept CI did not contain 0, indicating greater cross-band ISO coupling in PLV in TG LepR-Cre validation compared to WT discovery. (e) Spearman's correlations from WT validation vs. TG validation. The slope CI contained 1, and the intercept CI contained 0, indicating good agreement in terms of Spearman's correlation in TG validation compared to WT validation. (f) PLVs from WT validation vs. TG validation. The slope CI contained 1, but the intercept CI did not contain 0, indicating greater cross-band ISO coupling in PLV in TG LepR-Cre validation relative to WT validation.

**Figure 4:**
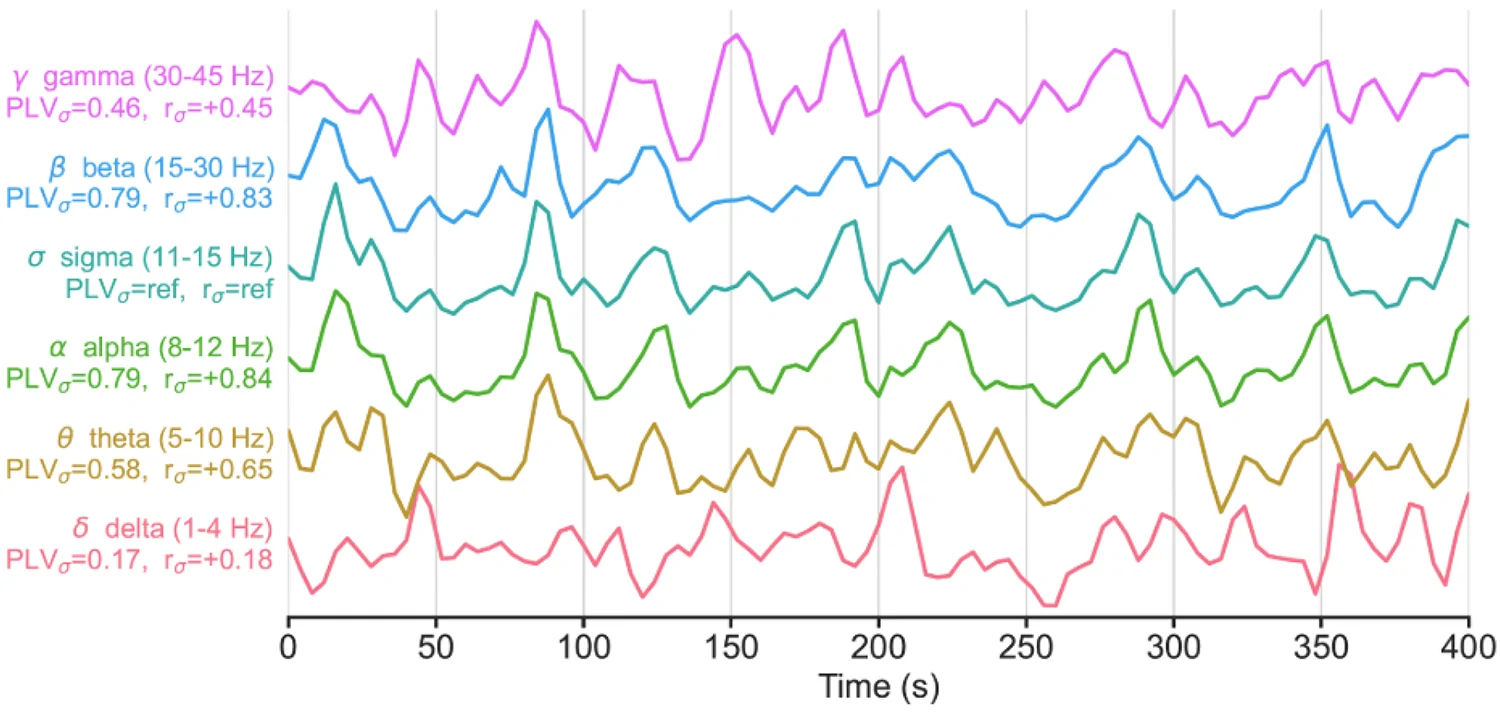
Illustration of infraslow oscillations across bands in a 400-second segment. The vertical gridlines are 50 seconds apart. The band power time series were band-pass filtered at 0.01-0.03Hz and z-scored for display (the amplitude axis is therefore omitted), based on the MSSV cohort, for which Spearman’s correlation (r) and PLV were closest to the cohort mean. The x-axis is time in seconds, and vertical gridlines mark every 50 seconds. The sigma ISO was used as the reference band. Relative to sigma, the coupling was strongest for alpha (r = +0.84, PLV = 0.79), beta (r = +0.83, PLV = 0.79), and theta (r = +0.65, PLV = 0.58), moderate for gamma (r = +0.45, PLV = 0.46), and weakest for delta (r = +0.18, PLV = 0.17).

**Figure 5:**
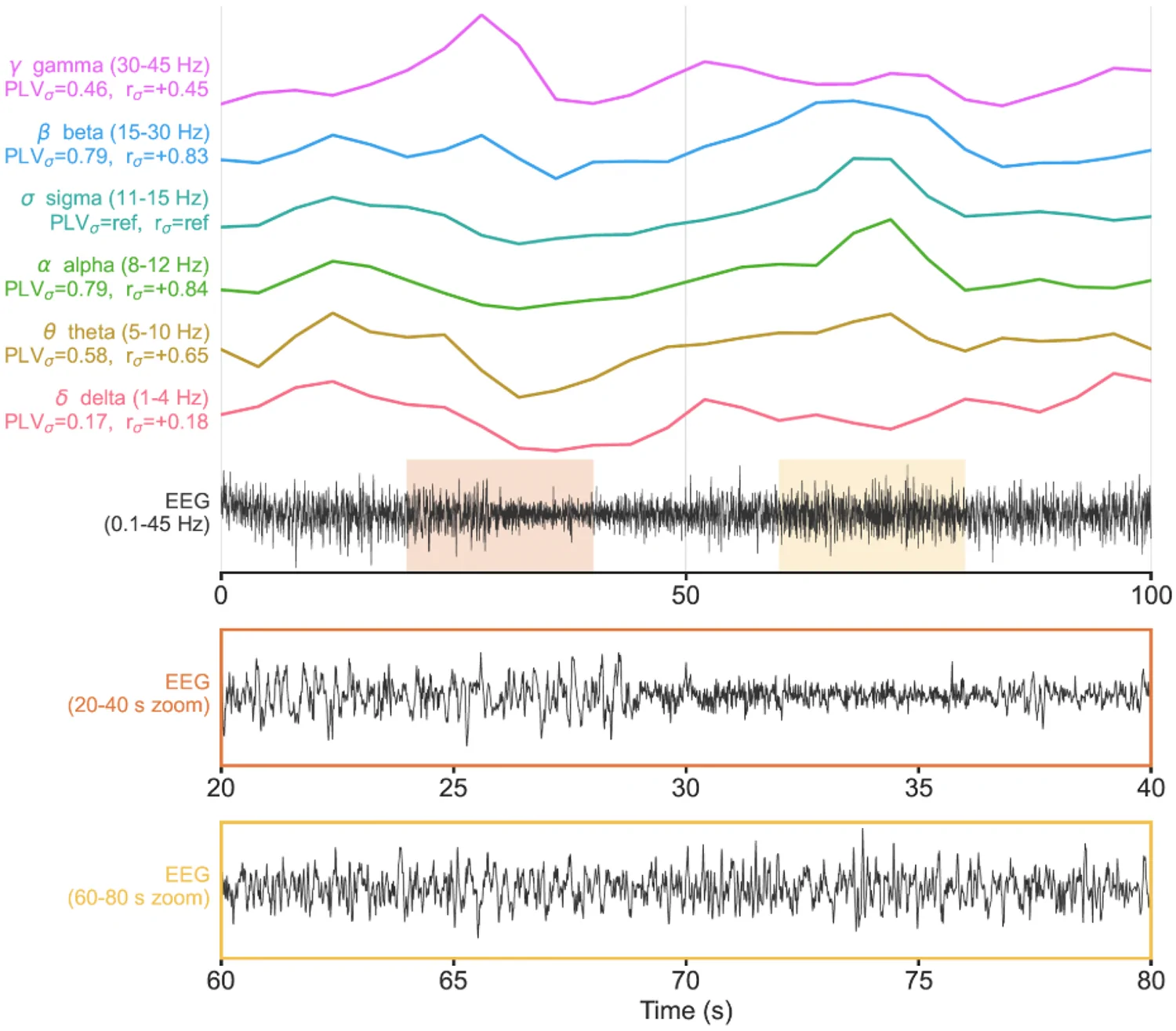
A zoom-in view of ISOs and EEG segments. The vertical gridlines in the top panel are 50 seconds apart. The two EEG panels at the bottom are labeled by colored bounding boxes to indicate the time of zoom-in windows of the same color. The EEG amplitude scale bar is omitted.

## Data Availability

The Mouse Sleep Staging Validation (MSSV) dataset is available at https://openneuro.org/datasets/ds006366/versions/1.0.1. The Oxford Mouse Polysomnography Benchmark Data Set is available at https://zenodo.org/records/10200482. The transgenic validation dataset is available upon reasonable request from the corresponding author.

## Statement of Significance

Infraslow oscillations (ISOs) are increasingly recognized as an important temporal pattern of NREM sleep, but prior work has focused primarily on the sigma band. Here, we show that ISOs extend across multiple EEG frequency bands and that alpha-, sigma-, beta-, and theta-band ISOs form a strongly coupled, replicated network across independent mouse sleep EEG datasets. These findings shift the view of the ISO from a band-specific phenomenon to a broadband, minute-scale temporal organization of NREM. This work may enable new investigations into how sleep microstructure coordinates neural activity, neuromodulation, memory processing, and glymphatic function, and how disruption of this organization may contribute to sleep and neurological disorders.

## Code Availability

The code is available at https://github.com/maxtan1/mouse-sleep-iso-analysis/.
